# Association of Project ECHO Training With Buprenorphine Prescribing by Primary Care Clinicians in Minnesota for Treating Opioid Use Disorder

**DOI:** 10.1001/jamahealthforum.2022.4149

**Published:** 2022-11-18

**Authors:** Anna R. Solmeyer, Aaron T. Berger, Sean L. Barton, Benjamin Nguyen, Gavin B. Bart, Brian Grahan, Heather J. Bell, Kurt M. DeVine, Weston Merrick

**Affiliations:** 1 Minnesota Management and Budget, St Paul; 2 Addiction Medicine Division, Hennepin Healthcare, Minneapolis, Minneota; 3 Addiction Services, CentraCare, St Cloud, Minnesota

## Abstract

**Importance:**

Buprenorphine is an approved medication for opioid use disorder (MOUD); however, prescribing buprenorphine is limited by a requirement to obtain a waiver to prescribe it (hereinafter, “DATA [Drug Abuse Treatment Act]–waiver”) and a lack of knowledge of the best practices among clinicians.

**Objective:**

To examine how Project ECHO (Extension for Community Healthcare Outcomes) telementoring is associated with changes in DATA-waiver attainment and buprenorphine prescribing among primary care clinicians in Minnesota.

**Design, Setting, and Participants:**

In this retrospective matched-cohort study of 918 clinicians, ECHO-trained clinicians were enrolled on the date they first attended ECHO (January 3, 2018, to June 11, 2020); comparison clinicians were assigned an enrollment date from the distribution of the first ECHO sessions. The baseline period was 12 months preceding enrollment, with follow-up for 18 months or until June 30, 2020. The ECHO-trained clinicians were a population-based sample of primary care clinicians who treated Medicaid patients in Minnesota 12 months prior to the initiation of ECHO training. This analysis used propensity score matching to select comparison clinicians who were similar across demographic and clinical practice characteristics at baseline in a 2:1 ratio. Follow-up was available for 167 ECHO-trained clinicians (54.6%) and 330 comparison clinicians (53.9%) at 18 months.

**Exposures:**

ECHO-trained clinicians attended at least 1 weekly, hour-long ECHO session. Comparison clinicians never participated in any ECHO sessions.

**Main Outcomes and Measures:**

DATA-waiver attainment, any buprenorphine prescribing, and the percentage of patients with opioid use disorder (OUD) who were prescribed buprenorphine.

**Results:**

The final sample included 918 clinicians (ECHO-trained [306]; comparison [612]), of whom 620 (67.5%) practiced outside the metropolitan Twin Cities (Minneapolis–St Paul) region. The mean (SD) age of the ECHO-trained clinicians was 46.0 (12.1) years and that of the comparison clinicians was 45.7 (12.3) years. Relative to the changes among the matched comparison clinicians, the ECHO-trained clinicians were more likely to obtain a DATA-waiver (difference-in-differences, 22.7 percentage points; 95% CI, 15.5-29.9 percentage points; *P* < .001) and prescribe any buprenorphine (16.5 percentage points; 95% CI, 10.4-22.5 percentage points; *P* < .001) after 6 quarters of follow-up. ECHO-trained clinicians prescribed buprenorphine to a greater share of patients with OUD (a difference of 7.6 percentage points per month; 95% CI, 4.6-10.6 percentage points per month; *P* < .001), relative to that prescribed by the comparison clinicians.

**Conclusions and Relevance:**

According to the findings of this matched-cohort study, ECHO telementoring may be associated with greater prescribing of buprenorphine by primary care clinicians. These findings suggest that Project ECHO training could be a useful tool for expanding access to MOUD.

## Key Points

**Question:**

What is the association between the training received by clinicians under Project ECHO (Extension for Community Healthcare Outcomes), a telementoring model that aims to expand the capacity for treating specific health conditions in primary care settings, and the number of clinicians obtaining a waiver needed to prescribe buprenorphine for treating opioid use disorder (OUD) and/or buprenorphine prescribing rates?

**Findings:**

In this matched-cohort study involving 918 clinicians, primary care clinicians who attended ECHO were more likely, by 23 percentage points, to obtain a waiver to prescribe buprenorphine than were the comparison clinicians not trained under ECHO. The ECHO-trained clinicians prescribed buprenorphine to 8 percentage points more of their patients with OUD than the number of patients prescribed by the matched comparison clinicians.

**Meaning:**

The findings of this study suggest that Project ECHO training may be a useful tool for expanding access to treatment for OUD.

## Teaser

This matched-cohort study examines the association between the training received by primary care clinicians in Minnesota under Project ECHO (Extension for Community Healthcare Outcomes) and the number of clinicians obtaining a waiver to prescribe buprenorphine for treating opioid use disorder.

## Introduction

Over the past 2 decades, the opioid use epidemic has emerged as one of the most pressing public health emergencies in the United States. Reported US opioid-related overdose deaths reached a high of more than 107 000 in the 12-month period ending February 2022.^1^ Three medications are approved for treating opioid use disorder (OUD): methadone, naltrexone, and buprenorphine. These medications for opioid use disorder (MOUDs) have proven highly effective in treating OUD and reducing mortality^2,3,4,5^; however, access to these treatments is limited. Whereas methadone can be obtained only through certified opioid treatment programs, naltrexone and buprenorphine can be prescribed in primary care settings.^6^ Given the challenges with naltrexone induction and nonadherence^7,8^ and the scarcity of programs that dispense methadone,^9^ buprenorphine is often the more accessible MOUD.^10^

Despite this, clinician capacity for buprenorphine is limited.^11^ Until 2021, clinicians were federally required to complete Drug Abuse Treatment Act of 2000 waiver (DATA-waiver) training. Although the training requirements were relaxed, clinicians are required to notify the Drug Enforcement Agency of their intention to prescribe buprenorphine and must complete the training if they intend to prescribe to more than 30 patients concurrently.^12^ Many waivered clinicians report not actively prescribing because of lack of training, concerns about diversion, lack of time for additional patients, and low reimbursement rates.^13,14^ There is, therefore, considerable interest in efforts to reduce these barriers and increase the capacity to prescribe buprenorphine.

This study examined the association of Project ECHO (Extension for Community Health Outcomes), a telementoring model that aims to expand the capacity for treating specific health conditions, such as OUD, with the number of clinicians attaining the DATA-waiver and prescribing buprenorphine. Each ECHO program consists of a “hub” where specialists work in an interdisciplinary team and “spokes” (typically clinicians in rural or underserved areas, or primary care clinicians who do not have specialized training in treating a particular illness), who connect to the hub through regular videoconferences for didactic and case-based learning.^15^ Studies of ECHO and ECHO-like programs for treating substance use disorders suggest that attending ECHO is associated with an increase in clinical knowledge,^16^ reduction in opioid prescribing,^17^ and increase in buprenorphine prescribing.^18^ Most existing research is descriptive and does not include comparison groups, leaving a gap in the knowledge about whether ECHO programs achieve their expected outcomes for patients.^19^

This study examined the association between attending either of 2 Minnesota-based ECHO hubs and the rates of both DATA-waiver attainment and buprenorphine prescribing among clinicians in the spokes. An earlier version of this study presented to the Minnesota state legislature^20^ was preregistered with Open Science Framework Registries.^21^ Compared with the report to the state legislature, the present report has been revised methodologically and focuses on the preregistered clinician-level outcomes regarding DATA-waiver attainment and buprenorphine prescribing.

## Methods

### Study Design

This retrospective matched-cohort study assessed the association between participation in Project ECHO telementoring and buprenorphine prescribing among a cohort of primary care clinicians who treat members of Minnesota Health Care Programs (MHCP), which provided publicly funded health insurance programs, from January 2017 to June 2020. The study was reviewed and approved by the Minnesota Department of Human Services Institutional Review Board. Informed consent was not required based on criteria under 45 CFR 46.116(d) because the study used only administrative data and posed minimal risk to the participants. The study followed the Strengthening the Reporting of Observational Studies in Epidemiology (STROBE) reporting guideline.^22^

The ECHO hubs provided attendance records for ECHO sessions from January 3, 2018, through June 11, 2020, including participants’ name, email, credentials, clinic address, and date(s) of attendance, collected through a web-based program management software called iECHO. We linked individuals in iECHO to Minnesota’s Medicaid Management Information System (MMIS) using fuzzy matching, which returned likely matches based on similarity in text across name, credential, and clinic address in entries in the 2 data sets. For entries with no likely matches or multiple potential matches, we attempted manual matching.

The overall cohort included active physicians, physician assistants, nurse practitioners, and clinical nurse specialists with primary clinician taxonomy codes indicating primary care (the eAppendix in the Supplement gives the taxonomy codes). We defined “active” clinicians as those with a practice in Minnesota, North Dakota, South Dakota, or Wisconsin and who treated at least 1 adult MHCP patient in the 12 months preceding their index date. Information on clinicians’ race and ethnicity was not available in the data set.

### Study Groups

The ECHO-trained group consisted of any eligible clinician who attended at least 1 ECHO telementoring session with 1 of 2 Minnesota ECHO hubs. Both hubs’ ECHO teams included physicians trained in addiction medicine. The hubs shared a primary aim to increase MOUD capacity among primary care clinicians and also provided education on other topics, including tapering high-dose opioid prescriptions, transitioning patients who were misusing prescription opioids to MOUDs, and other substances that can lead to overdose and death. Both hubs offered weekly, 1-hour telementoring sessions open to anyone who wished to attend, and participants could attend as many sessions as they liked. The index date for the ECHO-trained group was the date of their first telementoring session. Although the final quarter of the study overlapped with the beginning of the COVID-19 pandemic, the frequency of ECHO sessions did not change (eAppendix in the Supplement).

The comparison group was selected from among all qualifying clinicians who did not attend any ECHO session at either of the 2 ECHO hubs. We randomly assigned index dates to eligible comparison clinicians from the observed distribution of index dates among ECHO-trained clinicians of the same clinician type. Baseline characteristics and clinician type were used to match active comparison clinicians to ECHO-trained clinicians (the eAppendix in the Supplement gives the detailed screening procedure).

### Propensity Score Matching

We selected 2 comparison clinicians for each ECHO clinician by using an SAS (SAS Institute Inc) macro that implements N:1 propensity score matching.^23^ The propensity score model included both time-invariant characteristics and time-varying patient panel and clinical practice variables for each of the 4 quarters preceding the index date (the eAppendix in the Supplement gives the variables). Outcomes that occurred before the index date were included in the matching to address bias from nonparallel baseline trends.^24^ Comparison clinicians were matched to ECHO-trained clinicians within 0.2 SD of the logit of the propensity score.^25^

### Study Intervals

We aggregated outcomes into quarterly (3 continuous months) intervals during baseline and follow-up. The baseline period was defined as the 4 quarters prior to each clinician’s specific index date. Follow-up began on the index date and was terminated (1) after 6 quarters, (2) when the clinician’s enrollment to treat MHCP patients changed to “not active,” (3) when the clinician had no more MHCP patients in any subsequent study month, or (4) June 30, 2020.

### Primary Outcome: Buprenorphine Prescribing

This study’s main outcome was assessed through 3 measures of buprenorphine prescribing or capacity. The first measure was a dichotomous outcome for obtaining a DATA-waiver. ECHO-trained clinicians were linked individually by a National Provider Identifier number to an extract from the Controlled Substances Act Registry database that identified clinicians in Minnesota with a waiver to prescribe buprenorphine to at least 30 patients. The data were aggregated to the calendar quarter and available for calendar quarters 1 through 4 in 2015, quarter 1 in 2018, quarters 1 through 4 in 2019, and quarter 1 in 2020; it did not indicate the specific date when a clinician obtained a waiver. Because the study quarter and calendar quarter did not align, we took the conservative approach and assumed that the date the clinicians obtained a DATA-waiver was the last day of the calendar quarter in which their National Provider Identifier number was listed as having a waiver.

The second measure was a dichotomous outcome for prescribing buprenorphine to any patient with an OUD diagnosis. This was selected to separate obtaining a DATA-waiver from active use of a DATA-waiver. Buprenorphine prescriptions were identified by matching prescription fill claims in MMIS with buprenorphine National Drug Codes in the Healthcare Effectiveness Data and Information Set 2018-2020 (eTable 1 in the Supplement). Patients were classified as having an OUD if they had an *International Statistical Classification of Diseases and Related Health Problems, Tenth Revision (ICD-10)* diagnosis code for OUD (eTable 2 in the Supplement); for patients with a buprenorphine prescription, the diagnosis date must have occurred on or before the prescription date.

The third measure was the proportion of patients for whom the study clinician prescribed buprenorphine. The denominator was the number of unique patients with a diagnosed OUD who were treated by the clinician during each month (these patients may or may not have been prescribed buprenorphine). The numerator was the number of unique patients with a diagnosed OUD for whom the clinician prescribed buprenorphine during the month. We computed this proportion for each clinician over a monthly interval (because nearly all buprenorphine prescriptions were for ≤30 days) and then averaged the monthly proportions over quarterly intervals.

### Post Hoc Outcome: Number of Patients With OUD

We included a continuous outcome for the number of unique monthly patients with OUD seen by the clinician. This was chosen post hoc to identify if, in addition to changes in their prescribing practices, ECHO-trained clinicians also observed changes in the types of patients they saw, perhaps an increase in the number of patients with an OUD diagnosis. This outcome does not distinguish patients with preexisting OUD diagnoses who are newly treated by study clinicians from patients who were newly diagnosed with OUD. Rather, it provides additional context about changes in the denominator for the outcome measuring the proportion of patients with OUD who were prescribed buprenorphine.

### Effect Modifier: ECHO Attendance

As an exploratory analysis, we hypothesized that there would be a dose-response association between the number of sessions attended by each clinician and change in each outcome over time. We used iECHO records to measure the total ECHO attendance prior to the end of the follow-up period. We categorized attendance in thirds as 1 session (112 ECHO clinicians [36.6%]), 2 to 5 sessions (96 clinicians [31.4%]), and 6 or more sessions (98 clinicians [32.0%]). Comparison clinicians were assigned the number of sessions attended by their matched ECHO-trained clinician as their counterfactual level of exposure.

### Statistical Analysis

We used a difference-in-differences analysis to evaluate the association between ECHO and clinical practice over 18 months of follow-up. Data cleaning, univariate evaluation, and statistical analyses were completed in SAS, version 9.4. The main effects of interest are the overall type 3 test of fixed effects for the condition × time interaction terms estimated using linear mixed-effects models (continuous outcomes) or generalized linear mixed-effects models with a binomial distribution and identity link function (dichotomous outcomes) with random clinician-level intercepts and robust SEs. The condition × time interaction represents the difference-in-differences of outcomes between ECHO-trained and comparison clinicians. The reference period for all models, against which all changes are measured, was set as the last baseline quarter before the index date. Two-tailed *P* < .05 of the type 3 test of the interaction term indicated statistical significance.

The primary reason clinicians were lost to follow-up was administrative censoring at the end of the follow-up period (June 30, 2020). Owing to the matching on the index date, this censoring was both random and nondifferential with respect to the study group. Any observations missing at random were handled in continuous outcomes with maximum likelihood estimation, which provided unbiased parameter estimates.^26^

## Results

Of 1070 individuals in the iECHO attendance data, we linked 711 to MMIS clinician identifiers; we were unable to link the remaining 359 individuals because they were neither clinicians nor registered health care professionals with MHCP. Of the 711 individuals identified in MMIS, 390 were either not active clinicians or did not meet the type and taxonomy criteria. The remaining 321 ECHO participants met the study criteria (eFigure in the Supplement). From the 29 769 eligible comparison clinicians, 19 978 were active clinicians and 19 202 had baseline characteristics consistent with the observed values of ECHO-trained clinicians.

Propensity score matches were made for 306 ECHO clinicians (15 clinicians could not be matched), resulting in 612 comparison clinicians in the final sample. The mean (SD) age of the ECHO-trained clinicians was 46.0 (12.1) years and that of the comparison clinicians was 45.7 (12.3) years. ECHO attendance in the final sample ranged from 1 to 92 sessions, with a mean (SD) of 8.3 (14.5) sessions and a median of 2 sessions. The total number of clinicians included 563 physicians (61.3%), 258 nurse practitioners (28.1%), 5 certified nurse specialists (0.5%), and 92 physician assistants (10.0%). In the last baseline quarter, all cohort clinicians treated a mean (SD) of 69.9 (58.6) adult MHCP patients per month and 9.2 (14.0) MHCP patients with an OUD diagnosis per month. DATA-waivers were previously obtained by 147 clinicians (16.0%), and 126 clinicians (13.7%) prescribed buprenorphine at least once in the last baseline quarter. Importantly, baseline DATA-waiver attainment and buprenorphine prescribing rates were similar across the ECHO and comparison groups. There were no statistically significant differences in the baseline characteristics between the ECHO-trained and matched clinicians (Table 1). The mean (SD) follow-up period in both study groups was 4.4 (2.2) quarters; 167 (54.6%) of the ECHO-trained clinicians and 330 (53.9%) of the comparison clinicians had complete follow-up data.

**Table 1.** Baseline Characteristics of ECHO-Trained and Comparison Clinicians in Propensity Score–Matched Cohorts

Characteristic	Study group, No. (%)		Standardized difference^a^
	ECHO-trained clinicians (n = 306)	Comparison clinicians (n = 612)	
Age, mean (SD), y	46.0 (12.1)	45.7 (12.3)	0.03
Clinician type			
Physician	188 (61.4)	375 (61.3)	0.08
Nurse practitioner	85 (27.8)	173 (28.3)	
Clinical nurse specialist	3 (1.0)	2 (0.33)	
Physician assistant	30 (9.8)	62 (10.1)	
Region			
Minneapolis–St Paul metropolitan area	90 (29.4)	179 (29.3)	0.01
Rest of Minnesota	206 (67.3)	414 (67.7)	
Wisconsin, North Dakota, or South Dakota	10 (3.3)	19 (3.1)	
Unique adult MHCP patients per month, mean (SD), No.^b^	73.4 (61.1)	68.1 (57.3)	0.09
Unique adult MHCP patients per month with OUD diagnosis, mean (SD), No.^b^	9.7 (14.2)	8.9 (13.9)	0.06
Opioid analgesics prescribed per adult MHCP patient per month, mean (SD), MME^b^	50.3 (106.3)	48.2 (113.6)	0.02
Baseline DATA-waiver^b^	50 (16.3)	97 (15.9)	0.01
≥1 Buprenorphine prescription^b^	42 (13.7)	84 (13.7)	0
Quarters of follow-up	4.4 (2.2)	4.4 (2.2)	0

Abbreviations: DATA, Drug Abuse Treatment Act of 2000; ECHO, Extension for Community Health Outcomes; MHCP, Minnesota’s Health Care Programs; MME, morphine milligram equivalent; OUD, opioid use disorder.

^a^ Difference in means or proportions divided by SE.

^b^ Time-varying characteristics measured in the final quarter preceding clinician’s index date.

### Primary Analyses

**Figure 1.**
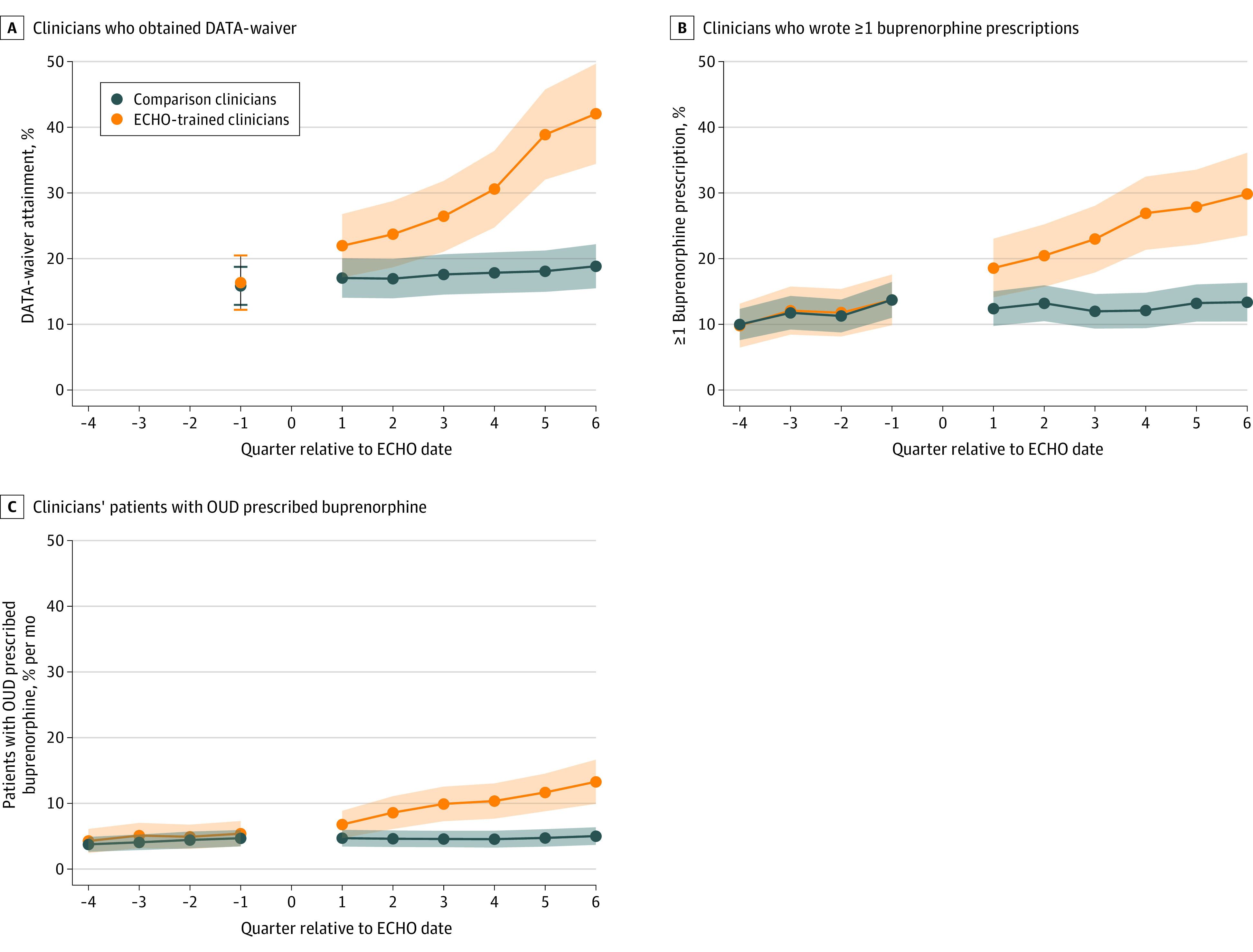
Main Outcomes Among Extension for Community Health Outcomes (ECHO)–Trained and Comparison Clinicians in a Propensity Score–Matched Cohort DATA indicates Drug Abuse Treatment Act of 2000; OUD, opioid use disorder.

DATA-waiver attainment increased significantly more among ECHO-trained clinicians than comparison clinicians, with difference-in-differences estimates of 4.4 percentage points (95% CI, 1.3-7.5 percentage points) after 1 quarter to 22.7 percentage points (95% CI, 15.5-29.9 percentage points) after 6 quarters (*P* < .001) (Table 2, Figure 1A; we explored an alternative specification for DATA-waiver status, given in the eAppendix and eTable 3 in the Supplement). As shown in Table 2, 42.0% of the clinicians who attended at least 1 ECHO session had obtained a DATA-waiver 18 months after beginning the ECHO training, compared with just 18.8% of the comparison clinicians. The dichotomous indicator for 1 or more buprenorphine prescriptions also increased faster among ECHO-trained prescribers, with difference-in-differences of 6.2 percentage points (95% CI, 3.2-9.1 percentage points) after 1 quarter to 16.5 percentage points (95% CI, 10.4-22.5 percentage points) after 6 quarters (*P* < .001) (Figure 1B). Compared with the change in comparison clinicians, after 1 quarter, ECHO-trained clinicians prescribed buprenorphine to 1.4 percentage points (95% CI, 0.45-2.3 percentage points) more of their monthly patients with OUD, and after 6 quarters to 7.6 percentage points (95% CI, 4.6-10.6 percentage points) more of such patients (*P* < .001) (Figure 1C). Intracluster correlation coefficients for each model are reported in eTable 4 in the Supplement.

**Table 2.** Means and Difference-in-Differences of Outcomes for ECHO-Trained and Comparison Clinicians in Propensity Score–Matched Cohorts

Relative quarter	Estimated mean (95% CI)^a^										*P* value^b^
	Baseline period				Follow-up period						
	−4	−3	−2	−1	1	2	3	4	5	6	
**DATA-waiver attainment, %^c^**											
ECHO-trained clinicians	NA	NA	NA	16.3 (12.2 to 20.5)	22.0 (17.1 to 26.8)	23.7 (18.7 to 28.8)	26.4 (21.1 to 31.8)	30.6 (24.8 to 36.4)	38.9 (32.0 to 45.7)	42.0 (34.4 to 49.7)	<.001
Comparison clinicians	NA	NA	NA	15.9 (13.0 to 18.7)	17.1 (14.1 to 20.1)	17.0 (14.0 to 19.9)	17.6 (14.5 to 20.7)	17.9 (14.8 to 21.0)	18.1 (15.0 to 21.2)	18.8 (15.5 to 22.2)	
Difference-in-differences	NA	NA	NA	1 [Reference]	4.4 (1.3 to 7.5)	6.3 (2.8 to 9.8)	8.4 (4.2 to 12.5)	12.2 (7.4 to 17.1)	20.3 (14.1 to 26.5)	22.7 (15.5 to 29.9)	
**≥1 Buprenorphine prescription, %**											
ECHO-trained clinicians	9.8 (6.5 to 13.1)	12.1 (8.4 to 15.8)	11.8 (8.2 to 15.4)	13.7 (9.9 to 17.6)	18.6 (14.1 to 23.0)	20.5 (15.7 to 25.2)	23.0 (17.9 to 18.0)	26.9 (21.4 to 32.5)	27.9 (22.2 to 33.5)	29.8 (23.6 to 36.1)	<.001
Comparison clinicians	10.0 (7.6 to 12.3)	11.8 (9.2 to 14.3)	11.3 (8.8 to 13.8)	13.7 (11.0 to 16.5)	12.4 (9.8 to 15.0)	13.2 (10.5 to 15.9)	12.0 (9.4 to 14.6)	12.1 (9.4 to 14.8)	13.2 (10.4 to 16.1)	13.4 (10.4 to 16.3)	
Difference-in-differences	−0.16 (−3.2 to 2.9)	0.33 (−2.6 to 3.3)	0.49 (−1.5 to 2.5)	1 [Reference]	6.2 (3.2 to 9.1)	7.3 (3.4 to 11.1)	11.0 (6.6 to 15.4)	14.8 (9.7 to 19.9)	14.6 (9.3 to 20.0)	16.5 (10.4 to 22.5)	
** Patients with OUD who were prescribed buprenorphine, % per mo**											
ECHO-trained clinicians	4.3 (2.4 to 6.1)	5.1 (3.2 to 7.0)	4.9 (3.1 to 6.8)	5.4 (3.5 to 7.3)	6.8 (4.7 to 8.9)	8.6 (6.1 to 11.1)	9.9 (7.3 to 12.5)	10.4 (7.7 to 13.0)	11.7 (8.8 to 14.5)	13.3 (9.9 to 16.6)	<.001
Comparison clinicians	3.8 (2.6 to 4.9)	4.1 (2.9 to 5.3)	4.4 (3.1 to 5.7)	4.7 (3.4 to 5.9)	4.7 (3.4 to 6.0)	4.6 (3.3 to 5.9)	4.6 (3.3 to 5.8)	4.5 (3.3 to 5.8)	4.7 (3.4 to 6.1)	5.0 (3.7 to 6.4)	
Difference-in-differences	−0.18 (−1.8 to 1.4)	0.33 (−1.1 to 1.8)	−0.22 (−1.4 to .92)	1 [Reference]	1.4 (0.45 to 2.3)	3.3 (1.4 to 5.2)	4.6 (2.6 to 6.7)	5.1 (2.9 to 7.3)	6.2 (3.8 to 8.7)	7.6 (4.6 to 10.6)	
**Unique patients with OUD per month, No.**											
ECHO-trained clinicians	8.5 (6.8 to 10.3)	8.7 (7.0 to 10.4)	9.1 (7.5 to 10.6)	9.7 (8.1 to 11.3)	10.5 (8.6 to 12.4)	11.3 (9.1 to 13.5)	11.3 (9.4 to 13.3)	11.1 (9.4 to 12.8)	11.6 (9.7 to 13.5)	12.3 (10.2 to 14.4)	.09
Comparison clinicians	7.7 (6.7 to 8.7)	8.0 (7.0 to 9.1)	8.5 (7.5 to 9.6)	8.9 (7.8 to 10.0)	9.2 (8.1 to 10.4)	9.3 (8.1 to 10.5)	8.9 (7.8 to 10.1)	9.0 (7.8 to 10.2)	9.0 (7.8 to 10.2)	9.3 (8.0 to 10.5)	
Difference-in-differences	0.03 (−1.1 to 1.2)	−0.15 (−1.0 to .76)	−0.28 (−0.76 to 0.20)	1 [Reference]	0.44 (−0.40 to 1.3)	1.2 (−0.09 to 2.4)	1.6 (0.42 to 2.7)	1.2 (−0.03 to 2.4)	1.8 (0.31 to 3.2)	2.2 (0.42 to 3.9)	

Abbreviations: DATA, Drug Abuse Treatment Act of 2000; ECHO, Extension for Community Health Outcomes; NA, not applicable; OUD, opioid use disorder.

^a^ Means and difference-in-differences were estimated with linear mixed-effects models (continuous outcomes) and generalized linear mixed-effects models (dichotomous outcomes) were estimated with clinician-level random intercepts.

^b^ Type 3 *P* value for exposure × time interaction.

^c^ Only 1 baseline DATA-waiver measure was computed owing to intermittent data availability prior to 2019.

### Post Hoc Analysis

Clinicians in each group treated similar numbers of patients with an OUD diagnosis in the baseline period. Following the index date, the number of unique patients with OUD per month increased more among ECHO-trained clinicians than comparison clinicians, with difference-in-differences from 0.44 unique patients per month (95% CI, −0.40 to 1.3 patients) after 1 quarter to 2.2 per month (95% CI, 0.42-3.9 patients) after 6 quarters (Table 2). However, the type 3 test of difference-in-differences was not statistically significant (*P* = .09).

### Effect Modification by Attendance

**Figure 2.**
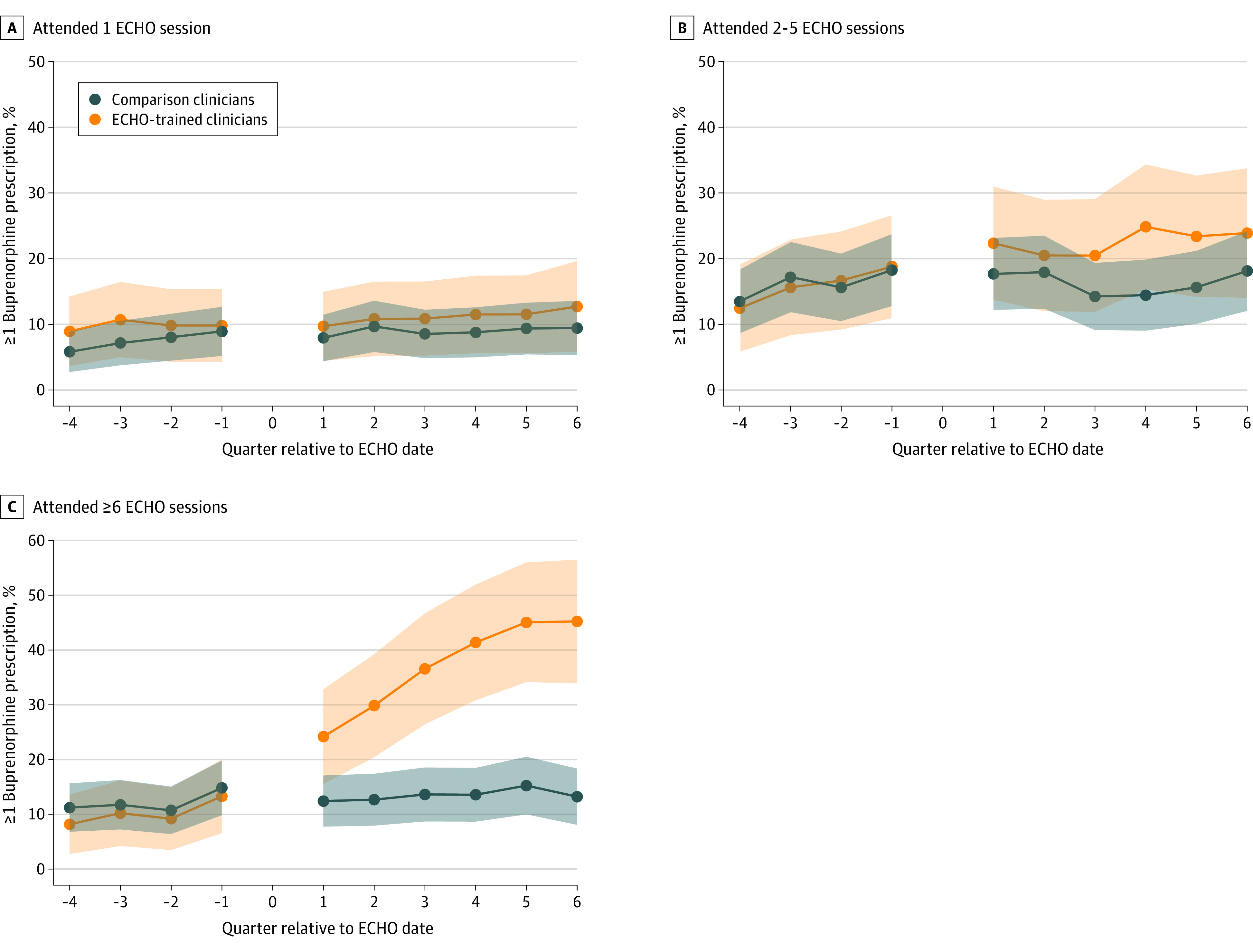
Association Between Project ECHO (Extension for Community Health Outcomes) Attendance and Writing 1 or More Buprenorphine Prescriptions Among ECHO-Trained and Comparison Clinicians in a Propensity Score–Matched Cohort

Attendance significantly modified associations between ECHO training and all outcomes (Table 3; Figure 2 illustrates the association between ECHO attendance and any buprenorphine prescribing). Compared with matched clinicians, difference-in-differences for clinicians who attended only 1 ECHO session did not indicate divergence over time in any clinical outcomes. ECHO-trained clinicians who attended 2 to 5 sessions had gradual increases in DATA-waiver attainment compared with their matched comparison clinicians, but no corresponding increases in buprenorphine prescribing or number of patients with OUD. Clinicians who attended 6 or more ECHO sessions had substantial, statistically significant increases in DATA-waiver attainment and buprenorphine prescribing and treated greater numbers of patients with OUD compared with their matched comparison cohort.

**Table 3.** Difference-in-Differences of Outcomes for ECHO-Trained Clinicians by Attendance Level and Comparison Clinicians in Propensity Score–Matched Cohorts

Relative quarter	Estimated difference-in-differences (95% CI)^a^										*P* value for interaction^b^
	Baseline period				Follow-up period						
	−4	−3	−2	−1	1	2	3	4	5	6	
**DATA-waiver attainment, %^c^**											
1 Session^d^	NA	NA	NA	1 [Reference]	1.5 (−1.9 to 4.8)	1.5 (−2.0 to 5.0)	3.2 (−1.6 to 8.0)	4.8 (−0.9 to 10.5)	6.8 (−1.8 to 15.3)	9.3 (−3.0 to 21.6)	.006
2-5 Sessions	NA	NA	NA	1 [Reference]	0.8 (−3.2 to 4.8)	3.4 (−2.0 to 8.7)	6.9 (−0.2 to 14.1)	9.3 (1.4 to 17.2)	11.1 (1.9 to 20.2)	13.9 (3.1 to 24.8)	
>6 Sessions	NA	NA	NA	1 [Reference]	10.2 (3.0 to 17.5)	13.1 (5.2 to 21.0)	13.5 (5.1 to 21.8)	20.4 (10.6 to 30.1)	35.9 (24.8 to 47.0)	35.1 (23.1 to 47.1)	
**≥1 Buprenorphine prescription, %**											
1 Session	2.2 (−1.8 to 6.2)	2.7 (−1.8 to 7.1)	0.9 (−0.3 to 2.1)	1 [Reference]	0.9 (−1.0 to 2.7)	0.2 (−3.3 to 3.8)	1.4 (−2.4 to 5.3)	1.8 (−2.5 to 6.2)	1.3 (−3.3 to 5.8)	2.4 (−3.6 to 8.3)	<.001
2-5 Sessions	−1.6 (−7.4 to 4.3)	−2.1 (−7.4 to 3.3)	0.5 (−3.1 to 4.2)	1 [Reference]	4.1 (−1.4 to 9.7)	2.0 (−4.4 to 8.4)	5.7 (−1.5 to 13.0)	9.9 (1.4 to 18.3)	7.2 (−1.2 to 15.6)	5.3 (−4.2 to 14.7)	
>6 Sessions	−1.5 (−7.7 to 4.6)	0 (−5.4 to 5.4)	0 (−5.0 to 5.0)	1 [Reference]	13.3 (6.7 to 19.9)	18.7 (10.4 to 27.0)	24.5 (15.2 to 33.8)	29.3 (19.1 to 39.6)	31.4 (20.5 to 42.2)	33.5 (22.4 to 44.7)	
** Patients with OUD (per month) who were prescribed buprenorphine, %**											
1 Session	1.0 (−1.4 to 3.4)	1.3 (−1.0 to 3.7)	0.1 (−1.2 to 1.4)	1 [Reference]	−0.1 (−0.8 to 0.6)	0.1 (−0.7 to 0.9)	−0.4 (−1.5 to 0.8)	0.1 (−1.7 to 1.8)	0.3 (−1.1 to 1.7)	1.0 (−1.4 to 3.4)	.005
2-5 Sessions	−1.5 (−4.2 to 1.3)	−0.6(−2.8 to 1.6)	−0.6 (−2.7 to 1.5)	1 [Reference]	0.6 (−1.0 to 2.1)	0.4 (−2.8 to 3.6)	1.5 (−1.2 to 4.2)	2.2 (−0.8 to 5.1)	1.9 (−1.2 to 5.1)	1.3 (−2.1 to 4.8)	
>6 Sessions	−0.7 (−3.6 to 2.3)	−0.4 (−3.1 to 2.3)	−0.6 (−3.0 to 1.8)	1 [Reference]	3.6 (1.4 to 5.8)	8.4 (4.2 to 12.7)	11.6 (6.9 to 16.2)	11.3 (6.5 to 16.1)	13.9 (8.6 to 19.3)	15.5 (9.8 to 21.3)	
**Unique OUD patients per month, count**											
1 Session	−0.6 (−2.0 to 0.7)	−0.7 (−1.8 to 0.4)	−0.4 (−1.3 to 0.6)	1 [Reference]	−0.5 (−1.2 to 0.2)	−0.5 (−1.5 to 0.5)	−0.9 (−1.9 to 0.2)	−1.9 (−3.9 to 0.16)	−1.9 (−3.9 to 0.1)	−2.6 (−5.5 to 0.3)	.004
2-5 Sessions	−0.7 (−2.1 to −0.7)	−0.5 (−1.9 to −0.9)	−0.4 (−1.1 to 0.4)	1 [Reference]	1.2 (−1.2 to 3.6)	2.3 (−1.4 to 6.0)	3.3 (0.4 to 6.3)	2.1 (−0.2 to 4.5)	2.4 (−0.4 to 5.3)	1.8 (−0.9 to 4.6)	
>6 Sessions	1.5 (−1.4 to 4.4)	0.8 (−1.3 to 2.9)	−0.1 (−0.9 to 0.7)	1 [Reference]	0.9 (0.003 to 1.7)	1.9 (0.7 to 3.2)	2.4 (0.9 to 4.0)	3.2 (1.3 to 5.1)	4.4 (2.1 to 6.7)	5.7 (2.8 to 8.6)	

Abbreviations: DATA, Drug Abuse Treatment Act of 2000; ECHO, Extension for Community Health Outcomes; NA, not applicable; OUD, opioid use disorder.

^a^ Difference-in-differences were estimated with linear mixed-effects models (continuous outcomes) and generalized linear mixed-effects models (dichotomous outcomes) were estimated with clinician-level random intercepts.

^b^ Type 3 *P* value for attendance × exposure × time interaction.

^c^ Only 1 baseline DATA-waiver measure was computed owing to intermittent data availability prior to 2019.

^d^ ECHO-trained clinicians in each attendance level compared with propensity score–matched comparison clinicians with no ECHO attendance.

## Discussion

In this study of a matched cohort of primary care clinicians, Project ECHO telementoring was found to be associated with greater prescribing of buprenorphine, an evidence-based OUD treatment. This study’s results showed a clear dose-response association with increasing attendance; those who attended at least 6 sessions had substantial increases in all outcomes. These findings support the use of Project ECHO to expand OUD treatment in primary care settings.

A key component of the study design was identifying a matched comparison cohort with parallel outcome trends through the 4 baseline quarters, lending strength to the conclusion that the observed associations were not the result of an unmeasured confounding variable. Specifically, we found that, of the clinicians who attended 1 or more ECHO sessions, 42.0% had obtained a DATA-waiver to prescribe buprenorphine 18 months after beginning ECHO training, compared with just 18.8% of the comparison clinicians. Moreover, while past studies have shown that many clinicians become waivered but never prescribe buprenorphine,^13,14^ we found that ECHO-trained clinicians also actively prescribed buprenorphine at a much higher rate than comparison clinicians at 18 months.

Importantly, our exploratory analyses suggest that clinicians may benefit most from attending more ECHO sessions. We found no statistically significant differences in clinical practice or patient panel for clinicians who attended between 1 and 5 ECHO sessions, but large differences emerged between clinicians who attended 6 or more ECHO sessions and the matched comparison clinicians. There is no minimum number of ECHO sessions that is considered “adequate” training; however, others have conducted similar exploratory analyses and found that higher attendance is associated with improved outcomes.^27^ This suggests that future research on program implementation could test approaches for retaining clinicians in ECHO training, such as by structuring the program to create “cohorts” of participants or offering a series of sessions focused on a specific topic or patient population.

### Limitations

This study has some limitations. A limitation of any observational study is the possibility that unmeasured differences, such as motivation to attend Project ECHO or treat patients with OUD, existed between the ECHO-trained and comparison clinicians. This limitation is more likely to affect the dose-response analysis. If clinicians who began prescribing more buprenorphine found that the sessions were of greater relevance to their practice, they may have been motivated to attend more sessions. The best way to avoid this kind of selection bias is to randomly assign clinicians to attend or not attend ECHO; however, this approach was not feasible in this study but could be a direction for future research. Efforts to promote attendance should also be evaluated for associations with both attendance and changes in clinical practice.

Furthermore, our access to medical claims data was limited to what was available in MMIS, which means we focused on Medicaid and other publicly funded health insurance programs. Although we were able to identify only the prescriptions that were filled and approved for reimbursement, this measure shows that ECHO telementoring was associated with increases in buprenorphine received by patients. Future work may be able to include clinicians and patients outside publicly funded health insurance programs to get a complete picture of ECHO’s impacts. In addition, compared with all primary care clinicians in MMIS, both the ECHO-trained and comparison clinicians in this study were more likely to already have a DATA-waiver, prescribe buprenorphine, and work with patients with OUD or other substance use disorders at baseline. Although we are confident that the comparison clinicians selected for this study were a good match for the ECHO-trained clinicians, our findings may not be generalizable to clinicians who do not currently see patients with OUD.

## Conclusions

This matched-cohort study’s findings suggest that Project ECHO telementoring is associated with greater prescribing of buprenorphine, an evidence-based OUD treatment. These findings support Project ECHO as a tool that may be useful for increasing access and capacity for MOUDs. Continued and expanded use of ECHO could be one important component in a robust continuum of care to mitigate the harm of opioids in our communities.

## Notes

Supplement. **eAppendix.** Supplemental Methods
eReference
**eTable 1.** National Drug Codes for Buprenorphine **eTable 2.** Diagnosis Codes Used to Identify Opioid Use Disorder **eTable 3.** Original and Alternative Specification for DATA-Waiver Attainment Among ECHO-Trained and Comparison Clinicians in a Propensity Score–Matched Cohort **eTable 4.** Intracluster Correlation Coefficients for Primary Mixed-Effects Models **eFigure.** Clinician Selection Flow Diagram
