# Error in Tables

**DOI:** 10.1001/jamahealthforum.2022.5308

**Published:** 2023-01-20

The Original Investigation titled “Association of Project ECHO Training With Buprenorphine Prescribing by Primary Care Clinicians in Minnesota for Treating Opioid Use Disorder,”^1^ published on November 18, 2022, was corrected to fix the presentation of the reference categories for difference-in-difference values in Tables 2 and 3. In all of those table cells, “1 [Reference]” should have appeared as “0 [Reference].” In addition, the last portion of footnote “a” in Tables 2 and 3 was modified slightly after the second closing parenthesis to read: “…all models were estimated with clinician-level random intercepts.” This article was corrected online.
