# Supplementary material for: Association of Project ECHO Training With Buprenorphine Prescribing by Primary Care Clinicians in Minnesota for Treating Opioid Use Disorder
Source: JAMA Health Forum. 2022 Nov 18;3(11):e224149. doi: 10.1001/jamahealthforum.2022.4149 (PMC9675001; doi:10.1001/jamahealthforum.2022.4149)
Supplement: Supplement. — eAppendix. Supplemental Methods eReference eTable 1. National Drug Codes for Buprenorphine eTable 2. Diagnosis Codes Used to Identify Opioid Use Disorder eTable 3. Original and Alternative Specification for DATA-Waiver Attainment Among ECHO-Trained and Comparison Clinicians in a Propensity Score–Matched Cohort eTable 4. Intracluster Correlation Coefficients for Primary Mixed-Effects Models eFigure. Clinician Selection Flow Diagram [file jamahealthforum-e224149-s001.pdf]

Supplementary Online Content

Solmeyer AR, Berger AT, Barton SL, et al. Association of Project ECHO training with buprenorphine prescribing by primary care clinicians in Minnesota for treating opioid use disorder. *JAMA Health Forum*. 2022;3(11):e224149. doi:10.1001/jamahealthforum.2022.4149

eAppendix. Supplemental Methods

eReference

eTable 1. National Drug Codes for Buprenorphine

eTable 2. Diagnosis Codes Used to Identify Opioid Use Disorder

eTable 3. Original and Alternative Specification for DATA-Waiver Attainment Among ECHO-Trained and Comparison Clinicians in a Propensity Score–Matched Cohort

eTable 4. Intracluster Correlation Coefficients for Primary Mixed-Effects Models

eFigure. Clinician Selection Flow Diagram

This supplementary material has been provided by the authors to give readers additional information about their work.

© Solmeyer AR et al. *JAMA Health Forum.*

eAppendix. Supplemental Methods

Clinician types and taxonomy codes

Clinicians were included if their NPI number was associated with clinician types 20 (physician), 65 (nurse practitioner), 68 (clinical nurse specialist), or 69 (physician assistant), and the first four characters of their primary clinician taxonomy codes included 207Q (family medicine), 207R (internal medicine), 207V (obstetrics and gynecology), 2080 (pediatrics), 2083 (preventive medicine), 2084 (psychology and neurology), 208D (general practice), 383A (physician assistant), 363L (nurse practitioner), or 364S (clinical nurse specialist).

Matching procedure

Initial screen

After collecting baseline characteristics of ECHO-trained clinicians and potential comparison clinicians, we screened out comparison clinicians whose baseline age or practice characteristics fell well outside the range observed for ECHO clinicians of the same clinician type. The screen was applied to ensure no comparison clinicians who were completely outside the values of the ECHO-trained clinicians. Age was bounded exactly (e.g., comparison physicians aged less than 29 or greater than 79 were excluded). Comparison clinicians were also excluded if their maximum or minimum value in any quarter of certain characteristics was outside of a buffered range of corresponding values among ECHO-trained clinicians of the same clinician type. The characteristics were: the count of unique patients per month, unique patients with prior substance use disorder diagnosis per month, unique patients with opioid use disorder (OUD) diagnosis per month, buprenorphine prescriptions per OUD patient per month, and opioid analgesic prescribing as morphine milligram equivalent (MME) per month per unique patient. The buffer was set as the floor or ceiling of the second significant digit of the minimum or maximum value for ECHO-trained clinicians. As an example, the maximum value of unique MHCP patients per month among ECHO-trained nurse practitioners (NPs) was 252; we screened out comparison NPs who treated more than 260 unique patients per month in any one quarter.

Propensity score matching

We fitted a propensity score model as a logistic regression including both time invariant covariates and time-varying patient panel and clinical practice variables. The time-varying covariates included measures of the outcomes of interest, included to reduce bias from pre-exposure deviations from parallel trends.[1]

Time invariant covariates included:

- Clinician type (physician, NP, PA, CNS)
- Index date (continuous)
- Age at index date (quintile)
- Geographic region (seven Minnesota regions, Wisconsin, North Dakota, South Dakota)
- Prior enrollment in a complementary training ("Buprenorphine Boot Camp," binary)
- Prior attainment of DATA-waiver (binary)

Time-varying covariates were measured in each of the four quarters preceding the index date. They included:

- Unique MHCP patients per month (none, up to 50th percentile, up to 75th percentile, > 75th percentile)
- Unique patients with OUD per month (none, up to 50th percentile, up to 75th percentile, > 75th percentile)
- Buprenorphine prescribing per OUD-diagnosed patient per month (none, up to 50th percentile of those who prescribed, > 50th percentile of those who prescribed)
- Opioid analgesic prescribing per MHCP patient per month (none, up to 50th percentile, up to 75th percentile, up to 90th percentile, up to 99th percentile, > 99th percentile)
- Any high-dose opioid analgesic prescribing >= 90 MME per day (binary)

© Solmeyer AR et al. *JAMA Health Forum.*

Sensitivity analyses

COVID-19 time period

The COVID-19 pandemic spanned the final quarter of our follow-up period (March –June 2020), which represented 10 percent of the full study period. During this time, there were substantial shifts in health care, such as most appointments moving from in-person to virtual, that could potentially change the patterns of ECHO attendance and the outcomes. To examine this possibility, we conducted a set of sensitivity checks.

ECHO sessions were offered with the usual frequency in March – June 2020. We examined the index dates for ECHO clinicians across quarters and found no difference between the number of clinicians whose first ECHO session date was between March and June 2020 and the number of clinicians whose first ECHO session date was during earlier quarters. This suggests that clinicians were starting ECHO at a similar rate before and during COVID.

We conducted a sensitivity analysis, presented in the legislative report (Appendix F), in which we removed data that was collected after March 1, 2020, and re-estimated the primary outcome models. Essentially, this tested what would have happened if the study period had ended on March 1, 2020 (before COVID-19) instead of June 11, 2020. Then, we visually compared plots of the estimated means over time and their 95% confidence intervals, for models with and without the COVID time period. None of the outcomes showed different patterns depending on whether the COVID time period was included or excluded.

Based on these results, we concluded that it was unlikely that including the one quarter of data during the beginning of the COVID-19 pandemic unduly influenced our findings.

Alternative specification of DATA-waiver status

Due to gaps in the availability of Controlled Substances Act Registry data, we tested an alternative specification for DATA-waiver. In this sensitivity analysis we defined DATA-waiver as either (1) confirmation in the Controlled Substances Act Registry or (2) having prescribed buprenorphine to any patient with OUD up to and including the quarter in question. The main analysis, which likely under-counts DATA-waivers, and the sensitivity analysis, which likely over-counts DATA-waivers, can be seen as bounding the true association between ECHO training and DATA-waiver attainment.

The association between ECHO training and change in DATA-waiver attainment in the alternate specification was consistent with the observed association in the original analysis (eTable 4). All difference-in-differences estimates in the sensitivity analysis fell within the 95% CI of the original analysis and vice versa.

eReference

1. Ryan AM. Well-Balanced or too Matchy–Matchy? The Controversy over Matching in Difference-in-Differences. *Health Services Research*. 2018;53(6):4106-4110. doi:10.1111/1475-6773.13015

© Solmeyer AR et al. *JAMA Health Forum.*

eTable 1. National Drug Codes for Buprenorphine

National Drug Code	Generic Product Name
00054017613	buprenorphine 2 mg sublingual tablet
00054017713	buprenorphine 8 mg sublingual tablet
00054018813	buprenorphine-naloxone 2 mg-0.5 mg sublingual tablet
00054018913	buprenorphine-naloxone 8 mg-2 mg sublingual tablet
00093215533	Buprenorphine 16 MG / Naloxone 4 MG Oral Strip
00093537856	buprenorphine 2 mg sublingual tablet
00093537956	buprenorphine 8 mg sublingual tablet
00093572056	buprenorphine-naloxone 2 mg-0.5 mg sublingual tablet
00093572156	buprenorphine-naloxone 8 mg-2 mg sublingual tablet
00228315303	buprenorphine 8 mg sublingual tablet
00228315309	Buprenorphine 8 MG Sublingual Tablet
00228315373	Buprenorphine 8 MG Sublingual Tablet
00228315403	buprenorphine-naloxone 2 mg-0.5 mg sublingual tablet
00228315409	buprenorphine-naloxone 2 mg-0.5 mg sublingual tablet
00228315473	buprenorphine-naloxone 2 mg-0.5 mg sublingual tablet
00228315503	buprenorphine-naloxone 8 mg-2 mg sublingual tablet
00228315509	buprenorphine-naloxone 8 mg-2 mg sublingual tablet
00228315567	Buprenorphine 8 MG / Naloxone 2 MG Sublingual Tablet
00228315573	buprenorphine-naloxone 8 mg-2 mg sublingual tablet
00228315603	buprenorphine 2 mg sublingual tablet
00228315609	Buprenorphine 2 MG Sublingual Tablet
00228315673	Buprenorphine 2 MG Sublingual Tablet
00247148701	1 ML Buprenorphine 0.3 MG/ML Injection
00378092393	buprenorphine 2 mg sublingual tablet
00378092493	buprenorphine 8 mg sublingual tablet
00378876716	Buprenorphine 8 MG / Naloxone 2 MG Oral Strip
00378876793	Buprenorphine 8 MG / Naloxone 2 MG Oral Strip
00378876816	Buprenorphine 12 MG / Naloxone 3 MG Oral Strip
00378876893	Buprenorphine 12 MG / Naloxone 3 MG Oral Strip
00406192303	Buprenorphine 2 MG / Naloxone 0.5 MG Sublingual Tabl
00406192309	Buprenorphine 2 MG / Naloxone 0.5 MG Sublingual Tabl
00406192403	Buprenorphine 8 MG / Naloxone 2 MG Sublingual Tablet
00406192409	Buprenorphine 8 MG / Naloxone 2 MG Sublingual Tablet
00406800503	Buprenorphine 2 MG / Naloxone 0.5 MG Sublingual Tabl
00406802003	buprenorphine-naloxone 8 mg-2 mg sublingual tablet
00409201203	1 ML Buprenorphine 0.3 MG/ML Cartridge
00409201232	1 ML Buprenorphine 0.3 MG/ML Cartridge
00517072501	1 ML Buprenorphine 0.3 MG/ML Injection
00517072505	1 ML Buprenorphine 0.3 MG/ML Injection
00781721606	Buprenorphine 2 MG / Naloxone 0.5 MG Oral Strip

© Solmeyer AR et al. *JAMA Health Forum.*

National Drug Code	Generic Product Name
00781721664	Buprenorphine 2 MG / Naloxone 0.5 MG Oral Strip
00781722706	Buprenorphine 4 MG / Naloxone 1 MG Oral Strip
00781722764	Buprenorphine 4 MG / Naloxone 1 MG Oral Strip
00781723806	Buprenorphine 8 MG / Naloxone 2 MG Oral Strip
00781723864	Buprenorphine 8 MG / Naloxone 2 MG Oral Strip
00781724906	Buprenorphine 12 MG / Naloxone 3 MG Oral Strip
00781724964	Buprenorphine 12 MG / Naloxone 3 MG Oral Strip
12496010001	buprenorphine 100 mg/0.5 mL subcutaneous solution, e
12496010002	0.5 ML Buprenorphine 200 MG/ML Prefilled Syringe
12496010005	0.5 ML Buprenorphine 200 MG/ML Prefilled Syringe
12496030001	buprenorphine 300 mg/1.5 mL subcutaneous solution, e
12496030002	1.5 ML Buprenorphine 200 MG/ML Prefilled Syringe
12496030005	1.5 ML Buprenorphine 200 MG/ML Prefilled Syringe
12496075701	1 ML Buprenorphine 0.3 MG/ML Injection
12496075705	1 ML Buprenorphine 0.3 MG/ML Injection
12496120201	buprenorphine-naloxone 2 mg-0.5 mg sublingual film
12496120203	buprenorphine-naloxone 2 mg-0.5 mg sublingual film
12496120401	buprenorphine-naloxone 4 mg-1 mg sublingual film
12496120403	buprenorphine-naloxone 4 mg-1 mg sublingual film
12496120801	buprenorphine-naloxone 8 mg-2 mg sublingual film
12496120803	buprenorphine-naloxone 8 mg-2 mg sublingual film
12496121201	buprenorphine-naloxone 12 mg-3 mg sublingual film
12496121203	buprenorphine-naloxone 12 mg-3 mg sublingual film
21695051510	1 ML Buprenorphine 0.3 MG/ML Cartridge
35356000407	Buprenorphine 8 MG / Naloxone 2 MG Sublingual Tablet
35356000430	Buprenorphine 8 MG / Naloxone 2 MG Sublingual Tablet
35356055530	Buprenorphine 2 MG Sublingual Tablet
35356055630	Buprenorphine 8 MG Sublingual Tablet
40042001001	1 ML Buprenorphine 0.3 MG/ML Injection
42023017901	1 ML Buprenorphine 0.3 MG/ML Injection
42023017905	1 ML Buprenorphine 0.3 MG/ML Injection
42023017910	1 ML Buprenorphine 0.3 MG/ML Injection
42291017430	buprenorphine-naloxone 2 mg-0.5 mg sublingual tablet
42291017530	buprenorphine-naloxone 8 mg-2 mg sublingual tablet
42858050103	buprenorphine 2 mg sublingual tablet
42858050203	buprenorphine 8 mg sublingual tablet
43063018407	Buprenorphine 8 MG / Naloxone 2 MG Sublingual Tablet
43063018430	Buprenorphine 8 MG / Naloxone 2 MG Sublingual Tablet
43063066706	buprenorphine 8 mg sublingual tablet
43063075306	buprenorphine 8 mg sublingual tablet
43598057901	Buprenorphine 2 MG / Naloxone 0.5 MG Oral Strip
43598057930	Buprenorphine 2 MG / Naloxone 0.5 MG Oral Strip

© Solmeyer AR et al. *JAMA Health Forum.*

National Drug Code	Generic Product Name
43598058001	Buprenorphine 4 MG / Naloxone 1 MG Oral Strip
43598058030	Buprenorphine 4 MG / Naloxone 1 MG Oral Strip
43598058101	Buprenorphine 12 MG / Naloxone 3 MG Oral Strip
43598058130	Buprenorphine 12 MG / Naloxone 3 MG Oral Strip
43598058201	Buprenorphine 8 MG / Naloxone 2 MG Oral Strip
43598058230	buprenorphine-naloxone 8 mg-2 mg sublingual film
47781035503	Buprenorphine 2 MG / Naloxone 0.5 MG Oral Strip
47781035511	Buprenorphine 2 MG / Naloxone 0.5 MG Oral Strip
47781035603	Buprenorphine 4 MG / Naloxone 1 MG Oral Strip
47781035611	Buprenorphine 4 MG / Naloxone 1 MG Oral Strip
47781035703	Buprenorphine 8 MG / Naloxone 2 MG Oral Strip
47781035711	Buprenorphine 8 MG / Naloxone 2 MG Oral Strip
47781035803	Buprenorphine 12 MG / Naloxone 3 MG Oral Strip
47781035811	Buprenorphine 12 MG / Naloxone 3 MG Oral Strip
47781071203	Buprenorphine 12 MG / Naloxone 3 MG Oral Strip
47781071211	Buprenorphine 12 MG / Naloxone 3 MG Oral Strip
49349055402	Buprenorphine 2 MG Sublingual Tablet
50090157100	Buprenorphine 8 MG Sublingual Tablet
50090292400	Buprenorphine 8 MG Sublingual Tablet
50268014411	Buprenorphine 2 MG / Naloxone 0.5 MG Sublingual Tabl
50268014415	buprenorphine-naloxone 2 mg-0.5 mg sublingual tablet
50268014511	Buprenorphine 8 MG / Naloxone 2 MG Sublingual Tablet
50268014515	buprenorphine-naloxone 8 mg-2 mg sublingual tablet
50268014550	Buprenorphine 8 MG / Naloxone 2 MG Sublingual Tablet
50383028733	Buprenorphine 8 MG / Naloxone 2 MG Sublingual Tablet
50383028793	buprenorphine-naloxone 8 mg-2 mg sublingual tablet
50383029433	Buprenorphine 2 MG / Naloxone 0.5 MG Sublingual Tabl
50383029493	buprenorphine-naloxone 2 mg-0.5 mg sublingual tablet
50383092493	buprenorphine 2 mg sublingual tablet
50383093093	buprenorphine 8 mg sublingual tablet
52125067802	Buprenorphine 2 MG Sublingual Tablet
52427069203	Buprenorphine 2 MG / Naloxone 0.5 MG Oral Strip
52427069211	Buprenorphine 2 MG / Naloxone 0.5 MG Oral Strip
52427069403	Buprenorphine 4 MG / Naloxone 1 MG Oral Strip
52427069411	Buprenorphine 4 MG / Naloxone 1 MG Oral Strip
52427069803	Buprenorphine 8 MG / Naloxone 2 MG Oral Strip
52427069811	Buprenorphine 8 MG / Naloxone 2 MG Oral Strip
52427071203	Buprenorphine 12 MG / Naloxone 3 MG Oral Strip
52427071211	Buprenorphine 12 MG / Naloxone 3 MG Oral Strip
52440010014	Buprenorphine 74.2 MG Drug Implant
53217013830	Buprenorphine 8 MG / Naloxone 2 MG Sublingual Tablet
53217024630	Buprenorphine 8 MG Sublingual Tablet

National Drug Code	Generic Product Name
53217032801	Buprenorphine 8 MG / Naloxone 2 MG Oral Strip
54123011430	buprenorphine-naloxone 11.4 mg-2.9 mg sublingual tab
54123090730	buprenorphine-naloxone 0.7 mg-0.18 mg sublingual tab
54123091430	buprenorphine-naloxone 1.4 mg-0.36 mg sublingual tab
54123092930	buprenorphine-naloxone 2.9 mg-0.71 mg sublingual tab
54123095730	buprenorphine-naloxone 5.7 mg-1.4 mg sublingual tabl
54123098630	buprenorphine-naloxone 8.6 mg-2.1 mg sublingual tabl
54569639900	Buprenorphine 8 MG / Naloxone 2 MG Oral Strip
54569640800	Buprenorphine 8 MG / Naloxone 2 MG Sublingual Tablet
54569657800	Buprenorphine 8 MG Sublingual Tablet
54868570700	Buprenorphine 8 MG / Naloxone 2 MG Sublingual Tablet
54868570701	Buprenorphine 8 MG / Naloxone 2 MG Sublingual Tablet
54868570702	Buprenorphine 8 MG / Naloxone 2 MG Sublingual Tablet
54868570703	Buprenorphine 8 MG / Naloxone 2 MG Sublingual Tablet
54868570704	Buprenorphine 8 MG / Naloxone 2 MG Sublingual Tablet
54868575000	Buprenorphine 2 MG / Naloxone 0.5 MG Sublingual Tabl
55154496200	Buprenorphine 2 MG Sublingual Tablet
55154496204	Buprenorphine 2 MG Sublingual Tablet
55154496206	Buprenorphine 2 MG Sublingual Tablet
55390010010	1 ML Buprenorphine 0.3 MG/ML Injection
55700014730	Buprenorphine 8 MG / Naloxone 2 MG Oral Strip
55700018430	Buprenorphine 2 MG / Naloxone 0.5 MG Sublingual Tabl
55700030230	Buprenorphine 2 MG Sublingual Tablet
55700030330	Buprenorphine 8 MG Sublingual Tablet
58118017608	Buprenorphine 2 MG Sublingual Tablet
58118017708	Buprenorphine 8 MG Sublingual Tablet
58118315608	Buprenorphine 2 MG Sublingual Tablet
58284010014	buprenorphine 74.2 mg subcutaneous implant
59385001201	buprenorphine-naloxone 2.1 mg-0.3 mg buccal film
59385001230	buprenorphine-naloxone 2.1 mg-0.3 mg buccal film
59385001401	buprenorphine-naloxone 4.2 mg-0.7 mg buccal film
59385001430	buprenorphine-naloxone 4.2 mg-0.7 mg buccal film
59385001601	buprenorphine-naloxone 6.3 mg-1 mg buccal film
59385001630	buprenorphine-naloxone 6.3 mg-1 mg buccal film
60429058611	Buprenorphine 2 MG / Naloxone 0.5 MG Sublingual Tabl
60429058630	buprenorphine-naloxone 2 mg-0.5 mg sublingual tablet
60429058633	Buprenorphine 2 MG / Naloxone 0.5 MG Sublingual Tabl
60429058711	Buprenorphine 8 MG / Naloxone 2 MG Sublingual Tablet
60429058730	buprenorphine-naloxone 8 mg-2 mg sublingual tablet
60429058733	Buprenorphine 8 MG / Naloxone 2 MG Sublingual Tablet
60846097003	Buprenorphine 2 MG / Naloxone 0.5 MG Sublingual Tabl
60846097103	Buprenorphine 8 MG / Naloxone 2 MG Sublingual Tablet

© Solmeyer AR et al. *JAMA Health Forum.*

National Drug Code	Generic Product Name
61786067802	Buprenorphine 2 MG Sublingual Tablet
61786091102	Buprenorphine 2 MG Sublingual Tablet
61786091202	Buprenorphine 8 MG Sublingual Tablet
62175045232	Buprenorphine 2 MG / Naloxone 0.5 MG Sublingual Tabl
62175045832	Buprenorphine 8 MG / Naloxone 2 MG Sublingual Tablet
62756045964	Buprenorphine 2 MG Sublingual Tablet
62756045983	buprenorphine 2 mg sublingual tablet
62756046064	Buprenorphine 8 MG Sublingual Tablet
62756046083	buprenorphine 8 mg sublingual tablet
62756096964	Buprenorphine 2 MG / Naloxone 0.5 MG Sublingual Tabl
62756096983	buprenorphine-naloxone 2 mg-0.5 mg sublingual tablet
62756097064	Buprenorphine 8 MG / Naloxone 2 MG Sublingual Tablet
62756097083	buprenorphine-naloxone 8 mg-2 mg sublingual tablet
63629402801	Buprenorphine 2 MG / Naloxone 0.5 MG Sublingual Tabl
63629402802	Buprenorphine 2 MG / Naloxone 0.5 MG Sublingual Tabl
63629403401	Buprenorphine 8 MG / Naloxone 2 MG Sublingual Tablet
63629403402	Buprenorphine 8 MG / Naloxone 2 MG Sublingual Tablet
63629403403	Buprenorphine 8 MG / Naloxone 2 MG Sublingual Tablet
63629403404	Buprenorphine 8 MG / Naloxone 2 MG Sublingual Tablet
63629409202	Buprenorphine 8 MG Sublingual Tablet
63629507401	Buprenorphine 8 MG / Naloxone 2 MG Sublingual Tablet
63629712501	Buprenorphine 2 MG Sublingual Tablet
63629712502	Buprenorphine 2 MG Sublingual Tablet
63629712503	Buprenorphine 2 MG Sublingual Tablet
63629712504	Buprenorphine 2 MG Sublingual Tablet
63629712505	Buprenorphine 2 MG Sublingual Tablet
63629712506	Buprenorphine 2 MG Sublingual Tablet
63629712507	Buprenorphine 2 MG Sublingual Tablet
63629712601	Buprenorphine 8 MG Sublingual Tablet
63629712602	Buprenorphine 8 MG Sublingual Tablet
63629712603	Buprenorphine 8 MG Sublingual Tablet
63629712604	Buprenorphine 8 MG Sublingual Tablet
63629712605	Buprenorphine 8 MG Sublingual Tablet
63629712606	Buprenorphine 8 MG Sublingual Tablet
63629712607	Buprenorphine 8 MG Sublingual Tablet
63629712608	Buprenorphine 8 MG Sublingual Tablet
63629712609	Buprenorphine 8 MG Sublingual Tablet
63629727001	Buprenorphine 2 MG / Naloxone 0.5 MG Sublingual Tabl
63629727002	Buprenorphine 2 MG / Naloxone 0.5 MG Sublingual Tabl
64725093003	Buprenorphine 8 MG Sublingual Tablet
64725093004	Buprenorphine 8 MG Sublingual Tablet
64725192403	Buprenorphine 2 MG Sublingual Tablet

© Solmeyer AR et al. *JAMA Health Forum.*

National Drug Code	Generic Product Name
64725192404	Buprenorphine 2 MG Sublingual Tablet
65162041503	buprenorphine-naloxone 8 mg-2 mg sublingual tablet
65162041509	Buprenorphine 8 MG / Naloxone 2 MG Sublingual Tablet
65162041603	buprenorphine-naloxone 2 mg-0.5 mg sublingual tablet
65162041609	Buprenorphine 2 MG / Naloxone 0.5 MG Sublingual Tabl
67046099030	Buprenorphine 2 MG Sublingual Tablet
67046099130	Buprenorphine 8 MG Sublingual Tablet
67046099230	Buprenorphine 2 MG Sublingual Tablet
67046099330	Buprenorphine 8 MG Sublingual Tablet
67046099430	Buprenorphine 2 MG Sublingual Tablet
67046099530	Buprenorphine 8 MG Sublingual Tablet
67046099630	Buprenorphine 2 MG Sublingual Tablet
67046099730	Buprenorphine 8 MG Sublingual Tablet
67046099830	Buprenorphine 2 MG Sublingual Tablet
67046099930	Buprenorphine 8 MG Sublingual Tablet
69189059101	Buprenorphine 2 MG / Naloxone 0.5 MG Sublingual Tabl
70022000110	1 ML Buprenorphine 0.3 MG/ML Injection
70518044200	Buprenorphine 8 MG Sublingual Tablet
70518065200	Buprenorphine 2 MG Sublingual Tablet
70518065201	Buprenorphine 2 MG Sublingual Tablet
70518065202	Buprenorphine 2 MG Sublingual Tablet
70518071100	Buprenorphine 2 MG Sublingual Tablet
70518071101	Buprenorphine 2 MG Sublingual Tablet
70518100700	Buprenorphine 2 MG / Naloxone 0.5 MG Sublingual Tabl
70518155700	Buprenorphine 2 MG Sublingual Tablet
70518162500	Buprenorphine 8 MG Sublingual Tablet
70518168400	Buprenorphine 8 MG / Naloxone 2 MG Sublingual Tablet
70518201400	Buprenorphine 8 MG Sublingual Tablet
70518221600	Buprenorphine 2 MG Sublingual Tablet
70518221700	Buprenorphine 2 MG Sublingual Tablet
70518221800	Buprenorphine 8 MG Sublingual Tablet
70518222600	Buprenorphine 2 MG Sublingual Tablet
71335035301	Buprenorphine 2 MG Sublingual Tablet
71335035302	Buprenorphine 2 MG Sublingual Tablet
71335035303	Buprenorphine 2 MG Sublingual Tablet
71335035304	Buprenorphine 2 MG Sublingual Tablet
71335035305	Buprenorphine 2 MG Sublingual Tablet
71335035306	Buprenorphine 2 MG Sublingual Tablet
71335035307	Buprenorphine 2 MG Sublingual Tablet
71335095001	Buprenorphine 2 MG Sublingual Tablet
71335095002	Buprenorphine 2 MG Sublingual Tablet
71335095003	Buprenorphine 2 MG Sublingual Tablet

© Solmeyer AR et al. *JAMA Health Forum.*

National Drug Code	Generic Product Name
71335095004	Buprenorphine 2 MG Sublingual Tablet
71335095005	Buprenorphine 2 MG Sublingual Tablet
71335095006	Buprenorphine 2 MG Sublingual Tablet
71335095007	Buprenorphine 2 MG Sublingual Tablet
71335115401	Buprenorphine 8 MG Sublingual Tablet
71335115402	Buprenorphine 8 MG Sublingual Tablet
71335115403	Buprenorphine 8 MG Sublingual Tablet
71335115404	Buprenorphine 8 MG Sublingual Tablet
71335115405	Buprenorphine 8 MG Sublingual Tablet
71335115406	Buprenorphine 8 MG Sublingual Tablet
71335115407	Buprenorphine 8 MG Sublingual Tablet
71335115408	Buprenorphine 8 MG Sublingual Tablet
71335115409	Buprenorphine 8 MG Sublingual Tablet
71335116301	Buprenorphine 8 MG Sublingual Tablet
71335116302	Buprenorphine 8 MG Sublingual Tablet
71335116303	Buprenorphine 8 MG Sublingual Tablet
71335116304	Buprenorphine 8 MG Sublingual Tablet
71335116305	Buprenorphine 8 MG Sublingual Tablet
71335116306	Buprenorphine 8 MG Sublingual Tablet
71335116307	Buprenorphine 8 MG Sublingual Tablet
71335116308	Buprenorphine 8 MG Sublingual Tablet
71335116309	Buprenorphine 8 MG Sublingual Tablet
76519117000	Buprenorphine 8 MG Sublingual Tablet
76519117001	Buprenorphine 8 MG Sublingual Tablet
76519117002	Buprenorphine 8 MG Sublingual Tablet
76519117003	Buprenorphine 8 MG Sublingual Tablet
76519117004	Buprenorphine 8 MG Sublingual Tablet
76519117005	Buprenorphine 8 MG Sublingual Tablet

© Solmeyer AR et al. *JAMA Health Forum.*

eTable 2. Diagnosis Codes Used to Identify Opioid Use Disorder

ICD-10 Diagnosis Code	Description
F11.1	Opioid abuse
F11.10	Opioid abuse, uncomplicated
F11.11	Opioid abuse, in remission
F11.12	Opioid abuse with intoxication
F11.120	Opioid abuse with intoxication, uncomplicated
F11.121	Opioid abuse with intoxication delirium
F11.122	Opioid abuse with intoxication with perceptual disturbance
F11.129	Opioid abuse with intoxication, unspecified
F11.13	Opioid abuse with withdrawal
F11.14	Opioid abuse with opioid-induced mood disorder
F11.15	Opioid abuse with opioid-induced psychotic disorder
F11.150	Opioid abuse with opioid-induced psychotic disorder with delusions
F11.151	Opioid abuse with opioid-induced psychotic disorder with hallucinations
F11.159	Opioid abuse with opioid-induced psychotic disorder, unspecified
F11.18	Opioid abuse with other opioid-induced disorder
F11.181	Opioid abuse with opioid-induced sexual dysfunction
F11.182	Opioid abuse with opioid-induced sleep disorder
F11.188	Opioid abuse with other opioid-induced disorder
F11.19	Opioid abuse with unspecified opioid-induced disorder
F11.2	Opioid dependence
F11.20	Opioid dependence, uncomplicated
F11.21	Opioid dependence, in remission
F11.22	Opioid dependence with intoxication
F11.220	Opioid dependence with intoxication, uncomplicated
F11.221	Opioid dependence with intoxication delirium
F11.222	Opioid dependence with intoxication with perceptual disturbance
F11.229	Opioid dependence with intoxication, unspecified
F11.23	Opioid dependence with withdrawal
F11.24	Opioid dependence with opioid-induced mood disorder
F11.25	Opioid dependence with opioid-induced psychotic disorder
F11.250	Opioid dependence with opioid-induced psychotic disorder with delusions
F11.251	Opioid dependence with opioid-induced psychotic disorder with hallucinations
F11.259	Opioid dependence with opioid-induced psychotic disorder, unspecified
F11.28	Opioid dependence with other opioid-induced disorder
F11.281	Opioid dependence with opioid-induced sexual dysfunction
F11.282	Opioid dependence with opioid-induced sleep disorder
F11.288	Opioid dependence with other opioid-induced disorder
F11.29	Opioid dependence with unspecified opioid-induced disorder

© Solmeyer AR et al. *JAMA Health Forum.*

ICD-10 Diagnosis Code	Description
F11.9	Opioid use, unspecified
F11.90	Opioid use, unspecified, uncomplicated
F11.92	Opioid use, unspecified with intoxication
F11.920	Opioid use, unspecified with intoxication, uncomplicated
F11.921	Opioid use, unspecified with intoxication delirium
F11.922	Opioid use, unspecified with intoxication with perceptual disturbance
F11.929	Opioid use, unspecified with intoxication, unspecified
F11.93	Opioid use, unspecified with withdrawal
F11.94	Opioid use, unspecified with opioid-induced mood disorder
F11.95	Opioid use, unspecified with opioid-induced psychotic disorder
F11.950	Opioid use, unspecified with opioid-induced psychotic disorder with delusions
F11.951	Opioid use, unspecified with opioid-induced psychotic disorder with hallucinations
F11.959	Opioid use, unspecified with opioid-induced psychotic disorder, unspecified
F11.98	Opioid use, unspecified with other specified opioid-induced disorder
F11.981	Opioid use, unspecified with opioid-induced sexual dysfunction
F11.982	Opioid use, unspecified with opioid-induced sleep disorder
F11.988	Opioid use, unspecified with other opioid-induced disorder
F11.99	Opioid use, unspecified with unspecified opioid-induced disorder

© Solmeyer AR et al. *JAMA Health Forum.*

eTable 3. Original and Alternative Specification for DATA-Waiver Attainment Among ECHO-Trained and Comparison Clinicians in a Propensity Score–Matched Cohort

	Estimated mean (95% confidence interval)[a]										
	Baseline period				Follow-up period						
Relative Quarter	-4	-3	-2	-1	1	2	3	4	5	6	P value[c]
DATA-waiver original specification (%)[b]											
ECHO-trained clinicians				16.3 (12.2, 20.5)	22.0 (17.1, 26.8)	23.7 (18.7, 28.8)	26.4 (21.1, 31.8)	30.6 (24.8, 36.4)	38.9 (32.0, 45.7)	42.0 (34.4, 49.7)	
Comparison clinicians				15.9 (13.0, 18.7)	17.1 (14.1, 20.1)	17.0 (14.0, 19.9)	17.6 (14.5, 20.7)	17.9 (14.8, 21.0)	18.1 (15.0, 21.2)	18.8 (15.5, 22.2)	
Difference-in-differences				(Reference)	4.4 (1.3, 7.5)	6.3 (2.8, 9.8)	8.4 (4.2, 12.5)	12.2 (7.4, 17.1)	20.3 (14.1, 26.5)	22.7 (15.5, 29.9)	<.001
DATA-waiver alternate specification (%)[b]											
ECHO-trained clinicians				19.6 (15.2, 24.1)	25.2 (20.2, 30.3)	28.3 (22.9, 33.6)	32.6 (26.8, 38.4)	36.4 (30.2, 42.5)	40.3 (33.7 47.0)	41.9 (34.7, 49.0)	
Comparison clinicians				17.8 (14.8, 20.8)	18.5 (15.4, 21.5)	19.6 (16.4, 22.7)	19.9 (16.7, 23.1)	20.5 (17.2, 23.8)	20.4 (17.1, 23.7)	21.0 (17.5, 24.4)	
Difference-in-differences				(Reference)	5.0 (2.0, 8.0)	6.9 (3.1, 10.7)	11.0 (6.3, 15.6)	14.0 (8.9, 19.2)	18.2 (12.2, 24.1)	19.1 (12.5, 25.7)	<.001

Abbreviations: DATA, Drug Abuse Treatment Act of 2000; ECHO, Extension for Community Health Outcomes

[a] Means and differences-in-differences estimated with linear mixed-effects models (continuous outcomes) and generalized linear mixed-effects models (dichotomous outcomes) with clinician-level random intercepts.

[b] Only one baseline DATA-waiver measure computed due to intermittent data availability prior to 2019. Alternate specification includes DATA-waiver record or history of any buprenorphine prescribing to patients with OUD.

[c] Type 3 P value for exposure * time interaction.

© Solmeyer AR et al. *JAMA Health Forum.*

eTable 4. Intracluster Correlation Coefficients for Primary Mixed-Effects Models[1]

Outcome	Intracluster correlation coefficient
DATA-waiver (%)	.569
1+ buprenorphine prescription (%)	.276
Percent of monthly OUD patients prescribed buprenorphine	.847
Count of unique OUD patients per month	.877

Abbreviations: DATA, Drug Abuse Treatment Act of 2000; OUD, opioid use disorder.
[1] See Table 2 in the main article for estimated means and 95% confidence intervals.

© Solmeyer AR et al. *JAMA Health Forum.*

eFigure. Clinician Selection Flow Diagram

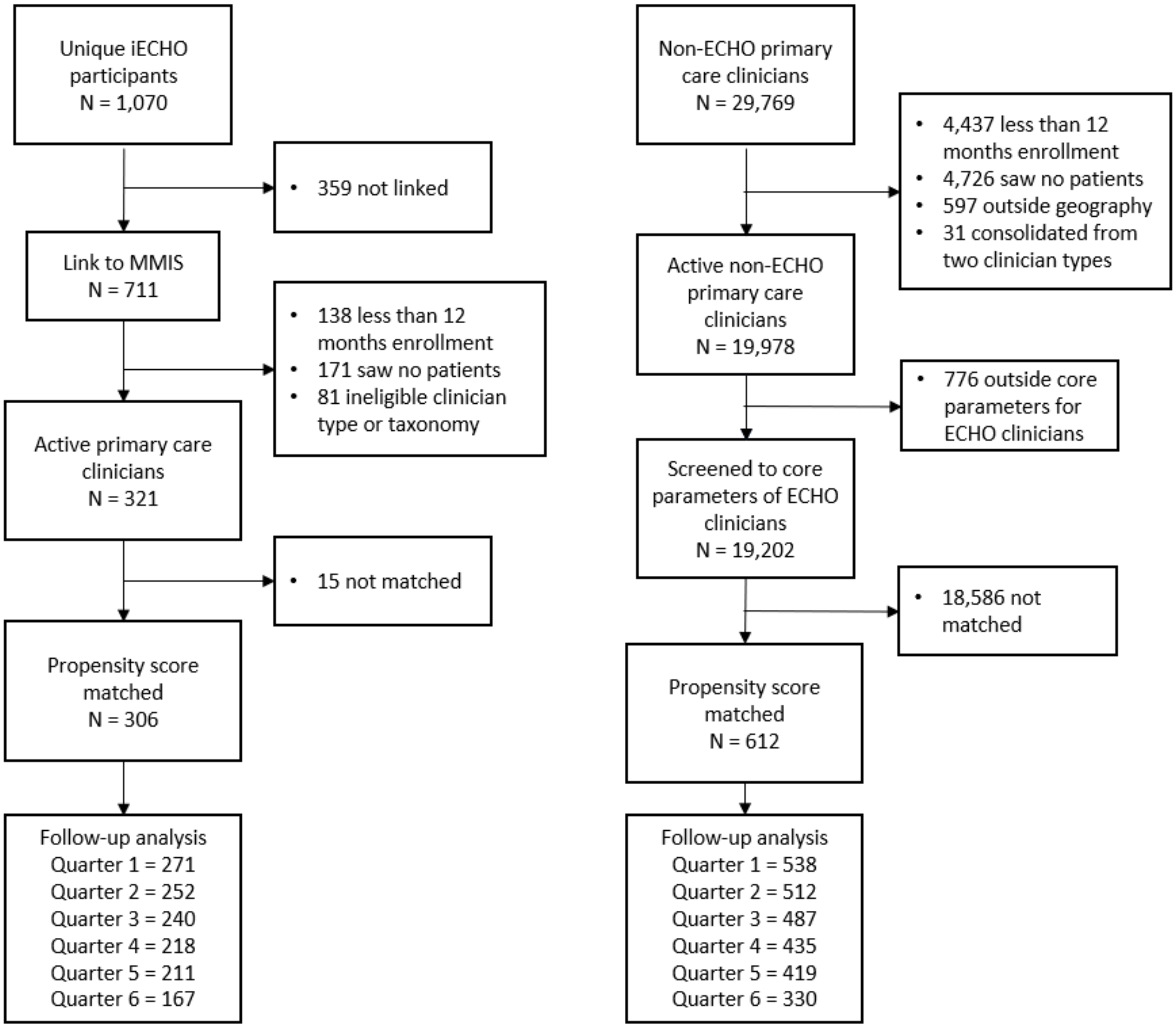

Abbreviation: ECHO, Extension for Community Health Outcomes

© Solmeyer AR et al. *JAMA Health Forum.*
